# The Paraventricular Thalamus as a Critical Node of Motivated Behavior *via* the Hypothalamic-Thalamic-Striatal Circuit

**DOI:** 10.3389/fnint.2021.706713

**Published:** 2021-06-18

**Authors:** Amanda G. Iglesias, Shelly B. Flagel

**Affiliations:** ^1^Neuroscience Graduate Program, University of Michigan, Ann Arbor, MI, United States; ^2^Michigan Neuroscience Institute, University of Michigan, Ann Arbor, MI, United States; ^3^Department of Psychiatry, University of Michigan, Ann Arbor, MI, United States

**Keywords:** paraventricular thalamus, lateral hypothalamus, nucleus accumbens, reward, motivation, associative learning, incentive salience

## Abstract

In this review, we highlight evidence that supports a role for the paraventricular nucleus of the thalamus (PVT) in motivated behavior. We include a neuroanatomical and neurochemical overview, outlining what is known of the cellular makeup of the region and its most prominent afferent and efferent connections. We discuss how these connections and distinctions across the anterior-posterior axis correspond to the perceived function of the PVT. We then focus on the hypothalamic-thalamic-striatal circuit and the neuroanatomical and functional placement of the PVT within this circuit. In this regard, the PVT is ideally positioned to integrate information regarding internal states and the external environment and translate it into motivated actions. Based on data that has emerged in recent years, including that from our laboratory, we posit that orexinergic (OX) innervation from the lateral hypothalamus (LH) to the PVT encodes the incentive motivational value of reward cues and thereby alters the signaling of the glutamatergic neurons projecting from the PVT to the shell of the nucleus accumbens (NAcSh). The PVT-NAcSh pathway then modulates dopamine activity and resultant cue-motivated behaviors. As we and others apply novel tools and approaches to studying the PVT we will continue to refine the anatomical, cellular, and functional definitions currently ascribed to this nucleus and further elucidate its role in motivated behaviors.

## Introduction

Behavioral neuroscience research has long been focused on unveiling the brain mechanisms underlying motivated behavior. Olds and Milner (1954) were among the first to identify brain structures involved in appetitive motivation and reinforcement learning. Their pioneering experiments showed that rats would repeatedly press a lever for electrical stimulation of brain regions that we now consider part of the mesocorticolimbic “reward” system. Since these initial experiments, however, we have learned that ascribing terms such as reward or reinforcement learning to a specific brain mechanism or circuit is overly simplistic both in terms of the semantics and the underlying neural mechanisms (Milner, 1991). We now know that the classic “motive circuit” extends beyond those brain structures first identified by Olds and Milner (1954; see also Milner, 1991), and more closely resembles that put forth by Ann Kelley in the early 2000s (Kelley et al., 2005).

**Figure 1:**
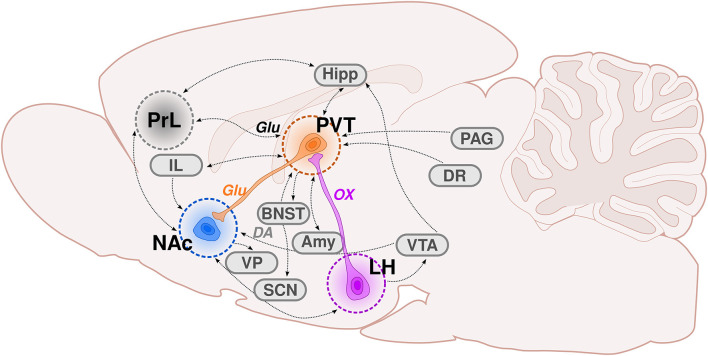
The PVT as a critical node of the hypothalamic-thalamic-striatal circuit. This graphic illustrates the afferents and efferents associated with the paraventricular nucleus of the thalamus (PVT), with an emphasis on the hypothalamic-thalamic-striatal circuit. The purple neuron represents orexinergic (OX) innervation from the lateral hypothalamus (LH)-PVT. The orange neuron is illustrating glutamatergic (Glu) innervation from the PVT-nucleus accumbens (NAc). The blue circle depicts the NAc, which receives substantial innervation from the PVT. The gray circle represents the anatomical location of the prelimbic cortex (PrL), which sends dense glutamatergic innervation to the PVT. The dotted black lines throughout the schematic depict neuronal connections, some of which are reciprocal (double arrow). Amy, amygdala; BNST, bed nucleus of the stria terminalis; DR, dorsal raphe; Hipp, hippocampus; IL, infralimbic cortex; LH, lateral hypothalamus; NAc, nucleus accumbens; PAG, periaqueductal gray; PrL, prelimbic cortex; PVT, paraventricular thalamic nucleus; SCN, suprachiasmatic nucleus; VP, ventral pallidum; VTA, ventral tegmental area; DA, dopamine; Glu, glutamate; OX, orexin.

Among the brain regions included on Ann Kelley’s maps of motivated behavior is the paraventricular nucleus of the thalamus (PVT). The PVT is a midline thalamic structure that is ideally placed to integrate information from arousal, limbic, cortical, and motor circuits in the brain (Figure 1). Not long after the initial studies by Olds and Milner (1954), Cooper and Taylor (1967) reported that rats with electrodes implanted within the PVT and surrounding areas demonstrated self-stimulation behavior. These findings were later supported by the work of Clavier and Gerfen (1982), who showed that levels of self-stimulation increased as electrodes became localized to midline thalamic structures, including the PVT. In the decade that followed, a number of studies demonstrated engagement of the PVT in response to drugs of abuse and associated stimuli (Deutch et al., 1995, 1998; Pierce and Kalivas, 1997; Young and Deutch, 1998; Stephenson et al., 1999). Despite this earlier research identifying a potential role for the PVT in reward processing and reinforcement learning, and Ann Kelley’s subsequent recognition of this nucleus as a primary node of motivated behavior, only in recent years has the PVT gained increasing attention in behavioral neuroscience research (see reviews: Martin-Fardon and Boutrel, 2012; James and Dayas, 2013; Haight and Flagel, 2014; Hsu et al., 2014; Matzeu et al., 2014; Kirouac, 2015; Millan et al., 2017; Matzeu and Martin-Fardon, 2018; Zhou and Zhu, 2019; Barson et al., 2020; McGinty and Otis, 2020; Curtis et al., 2021; McNally, 2021).

Here, we will review the literature that supports an integrative role of the PVT in motivated behavior as first put forth by Kelley and colleagues (Kelley et al., 2005). We will focus specifically on the hypothalamic-thalamic-striatal axis, with a proposed role for the PVT in encoding signals from the lateral hypothalamus (LH) and, in turn, sending signals to the ventral striatum to guide motivated behavior. We will highlight anatomical, pharmacological, and behavioral studies that have aimed to unmask the function of this circuit. Further, we include research from our own laboratory which has revealed a specific role for the PVT in encoding the incentive motivational value of reward cues.

## Neuroanatomical and Neurochemical Characteristics of The Paraventricular Nucleus of The Thalamus

Comprehensive tracing and anatomical studies have delineated the afferent and efferent connections of the PVT (see Figure 1), as well as the neurotransmitter and neuropeptide profiles within the region (for review see, Kirouac, 2015; Barson et al., 2020; Curtis et al., 2021). The PVT is densely innervated by glutamatergic afferents from the medial prefrontal cortex, with the most abundant population coming from the prelimbic cortex (PrL; Li and Kirouac, 2012). Although less dense relative to cortical inputs, the PVT also receives afferents from a range of subcortical regions including the hypothalamus, LH, hippocampus, and amygdala; and from brainstem structures including the periaqueductal gray and dorsal raphe (Van der Werf et al., 2002; Vogt et al., 2008; Hsu and Price, 2009; Li and Kirouac, 2012). The PVT has a number of reciprocal connections, with primarily glutamatergic efferents terminating in both cortical and subcortical structures including the PrL, hypothalamus, hippocampus, and amygdala (Su and Bentivoglio, 1990; Li and Kirouac, 2008, 2012; Vertes and Hoover, 2008). The PVT also sends glutamatergic efferents to the nucleus accumbens core (NAcC) and shell (NAcSh; Parsons et al., 2006; Vertes and Hoover, 2008; Dong et al., 2017), making it ideally positioned to integrate information regarding internal states and the external environment and translate it into motivated actions (Kelley et al., 2005).

As its afferents and efferents are primarily glutamatergic, it is not surprising that glutamatergic markers, like vesicular glutamate transporter 2 (vGLUT2) mRNA, are highly expressed in the PVT (Barroso-Chinea et al., 2007). However, the neurochemical composition of the PVT extends beyond glutamate and is complex and heterogeneous. Other neurotransmitter and neuropeptide systems that have been observed within this thalamic nucleus include dopamine (DA), gamma aminobutyric acid (GABA), opioids, cocaine-and amphetamine-regulated transcript (CART), and orexin (Lindvall et al., 1984; Koylu et al., 1997, 1998; Peyron et al., 1998). Through retrograde labeling of tyrosine hydroxylase (TH) neurons, it was shown that the majority of DA fibers in the PVT originate in the hypothalamus and periaqueductal gray (Li et al., 2014). Of the dopamine receptors, the D3 receptor, known to play a role in drug-seeking behavior (Xi et al., 2006; Peng et al., 2009; Khaled et al., 2010; Higley et al., 2011; Rice et al., 2013), appears to be the most abundant in the PVT (Mansour and Watson, 1995; Haight and Flagel, 2014). GABAergic neurons seem to be lacking within the PVT (Ottersen and Storm-Mathisen, 1984; Feldblum et al., 1993; see also Alamilla and Aguilar-Roblero, 2010). However, the PVT receives dense GABAergic innervation from several brain regions, including the reticular nucleus of the thalamus, the brainstem, and a number of hypothalamic nuclei (Cornwall and Phillipson, 1988; Chen and Su, 1990; Krout et al., 2002; Zhang et al., 2006; Li and Kirouac, 2012). That arising from the LH (Otis et al., 2019) and the zona incerta (Zhang and van den Pol, 2017) seems to be particularly important for motivated behaviors. Several neuropeptide systems are also abundant in the PVT. From the opioid family, expression of dynorphins and enkephalins are apparent, as are the kappa, mu, and delta-opioid receptors (Marchant et al., 2010; Curtis et al., 2021). CART-containing neuron fibers are also found throughout the PVT (Koylu et al., 1998; Kirouac et al., 2006), and have been implicated in drug-seeking behavior (Dayas et al., 2008; James et al., 2010; Choudhary et al., 2018). Similarly, orexin signaling within the PVT has been shown to play a role in drug-seeking and other reward-related behaviors (Choi et al., 2010, 2012; Matzeu et al., 2016; Meffre et al., 2019; Matzeu and Martin-Fardon, 2020, 2021), with both isoforms of orexin and the associated orexin receptors apparent throughout the nucleus (Marcus et al., 2001).

The PVT is often divided into anterior (aPVT) and posterior (pPVT) subregions based on anatomical boundaries that correspond with observational differences in cellular organization and function. The entire axis of the PVT is tightly linked to the limbic system *via* both afferent and efferent connections (Li and Kirouac, 2008, 2012; Kirouac, 2015), and some to a different degree in the aPVT vs. pPVT. Compared to the pPVT, the aPVT receives more dense neuronal projections from areas including the hypothalamus, ventral hippocampal subiculum, and infralimbic cortex (Canteras and Swanson, 1992; Li and Kirouac, 2012); and has reciprocal connections with a number of brain regions, including the suprachiasmatic nucleus (Moga and Moore, 1997; Novak et al., 2000; Li and Kirouac, 2008; Vertes and Hoover, 2008). These connections support the proposed role of the aPVT in reward-seeking behavior (Do-Monte et al., 2017; Cheng et al., 2018) and arousal (Kolaj et al., 2012; Gao et al., 2020). The pPVT is more heavily innervated by neurons from the hypothalamus and prelimbic, infralimbic, and insular cortices (Li and Kirouac, 2012), and sends more projections to the bed nucleus of the stria terminalis and central nucleus of the amygdala (Li and Kirouac, 2008). These neuroanatomical connections support a proposed role for the pPVT in stress-responsivity (Bhatnagar and Dallman, 1998, 1999; Bhatnagar et al., 2002, 2003; Heydendael et al., 2011) and anxiety-like behaviors (Li et al., 2010a,b; Barson and Leibowitz, 2015). Both the aPVT and pPVT project to the nucleus accumbens (NAc), with the densest projections to the NAcSh (Li and Kirouac, 2008; Vertes and Hoover, 2008). The aPVT projects more heavily to the dorsomedial NAcSh, and the pPVT to the ventromedial NAcSh (Dong et al., 2017). In line with the presumed functional distinctions between the aPVT and pPVT, the dorsomedial NAcSh has been implicated in positive emotional valence (e.g., appetitive and reward-related behaviors; Peciña et al., 2006; Reed et al., 2015), whereas the ventromedial NAcSh has been implicated in encoding negative emotional valence (e.g., defensive behaviors; Reynolds and Berridge, 2008; Berridge and Kringelbach, 2015). Further, as comprehensively outlined in a recent review article (Curtis et al., 2021), neuropeptides involved in arousal (e.g., galanin and proenkephalin) are apparent in the aPVT, while those involved in depressive- and anxiety-like behaviors (e.g., tachykinin 2, cholecystokinin, corticotropin-releasing hormone) are apparent in the pPVT. Neuropeptides associated with reward-related behaviors (e.g., tachykinin 2 and CART) are most abundant in the middle portion of the PVT (for review see Curtis et al., 2021). While much of the existing literature focuses on aPVT vs. pPVT, new technologies are pushing many in the field to study this nucleus based on molecular and cellular indicators. For example, recent work in mice identified two major classes of PVT neurons based on genetic markers (Gao et al., 2020). These Type I (*Drd2*) and Type II (*Gal*) PVT neurons were shown to differ in anatomy and function, representing a novel approach to characterizing the PVT along anterodorsal and posteroventral gradients (Gao et al., 2020). Anatomical, cellular, and functional characterization of the PVT is complex, often ambiguous, and sometimes conflicting; yet, there is clearly a role for this nucleus in motivated behaviors. If one were to assign functions based on anatomical boundaries and connectivity, the aPVT appears to play a predominant role in positive emotional valence and appetitive motivation (Barson et al., 2015; Do-Monte et al., 2017; Cheng et al., 2018), whereas the pPVT may be more involved in negative emotional valence and aversive motivation (Bhatnagar and Dallman, 1998, 1999; Bhatnagar et al., 2002, 2003; Li et al., 2010a,b; Heydendael et al., 2011; Barson and Leibowitz, 2015).

## The PVT as A Critical Node of The Hypothalamic-Thalamic-Striatal Circuit

The PVT has been postulated to mediate motivated behavior *via* its placement in the hypothalamic-thalamic-striatal circuit (Kelley et al., 2005). Kelley and colleagues recognized that the PVT is a key node of this subcortical circuit, acting to encode information about energy and arousal states from the hypothalamus while sending this information to the striatal complex to guide motivated behavior. Specifically, it was hypothesized that the PVT integrates orexinergic input from the LH and, in turn, sends information *via* glutamatergic projections to the nucleus accumbens to elicit actions (see Figure 1). These glutamatergic terminals from the PVT interface with DA neurons in the NAcSh (Pinto et al., 2003) and can alter DA release, independent of the VTA (Parsons et al., 2007). Despite these provocative neuroanatomical and neurochemical findings, relatively little behavioral neuroscience research has focused on the LH-PVT-NAc circuit; but that which has supports its proposed role in motivated behaviors.

The LH, the primary source of orexin neurons in the brain, was originally studied for its role in feeding behaviors (Anand and Brobeck, 1951; Hoebel and Teitelbaum, 1962), and subsequently in other homeostatic functions, such as sleep regulation and circadian rhythms (Kolaj et al., 2007; Colavito et al., 2015). The two orexin peptides, orexin-A, and orexin-B, are synthesized from the same mRNA transcript, prepro-orexin (de Lecea et al., 1998), found in the LH (Tsujino and Sakurai, 2013). Orexinergic neurons in the LH project to a wide variety of areas, including the cerebral cortex, hippocampus, and thalamus, with a large percentage terminating in the PVT (Peyron et al., 1998; Kirouac et al., 2005). The density of LH orexinergic input is stronger in the pPVT compared to aPVT (Goto and Swanson, 2004; Kirouac et al., 2005). It is known that orexin is excitatory (de Lecea et al., 1998), orexinergic neurons can co-release glutamate (Torrealba et al., 2003; Schöne et al., 2014), and that LH orexinergic inputs can have an excitatory effect on postsynaptic neurons in the PVT (Ishibashi et al., 2005; Huang et al., 2006). It should be noted, however, that a recent report suggests that LH-PVT neurons are primarily GABAergic (Otis et al., 2019). This finding is based largely on electrophysiological recordings, and possibly due to the relatively low numbers of PVT-projecting orexinergic neurons (Kirouac et al., 2005), which would preclude the ability to detect an electrophysiological signature of these neurons. Indeed, there is likely a disconnect between electrophysiological evidence and function, as there are several studies demonstrating that orexinergic signaling within the PVT plays a predominant role in appetitive motivated behaviors (e.g., Li et al., 2009; Stratford and Wirtshafter, 2013; Barson et al., 2015). Notably, stimulation of GABAergic neurons in the LH-PVT pathway also elicits consummatory and reward-seeking behaviors (Wu et al., 2015; Zhang and van den Pol, 2017). Given the characteristics and temporal dynamics of GABA- vs. orexinergic signaling, the influence of these systems on PVT function and motivated behaviors likely occurs *via* distinct mechanisms, which warrant further investigation.

The orexin peptides, and their two G-protein coupled receptors, orexin-1 (OX1) and orexin-2 (OX2) (Sakurai et al., 1998), are found throughout the PVT (Marcus et al., 2001; Parsons et al., 2006). Each receptor has a different binding affinity for each neuropeptide, with OX-1 having a higher affinity for orexin-A, and OX-2 having similar affinities for orexin-A and orexin-B (Marcus et al., 2001). Orexin signaling within the PVT has been implicated in behavioral responses to both natural rewards and drugs of abuse (Li et al., 2009; Stratford and Wirtshafter, 2013; Barson et al., 2015). For example, microinjections of orexin-A into the PVT elicits food- and drug-seeking behavior (Barson et al., 2015; Matzeu et al., 2018); whereas antagonism of OX-1 in the PVT decreases cue-induced reward-seeking behavior (Cole et al., 2015). Other reports have demonstrated the involvement of OX-2 (Matzeu et al., 2016), but not OX-1 (James et al., 2011), receptors in the PVT in cocaine-seeking behavior. Regardless of the mechanism, these findings highlight comparable roles for both the LH-PVT pathway and orexin signaling within the PVT in motivated behaviors (Barson et al., 2015; Matzeu et al., 2016, 2018).

Given the data reviewed above, it is perhaps not surprising that manipulations of the orexin system within the PVT are known to impact DA transmission in the NAc, a critical component of reward processing. For example, *in vivo* administration of orexin-A into the PVT significantly increases dopamine levels in the NAc (Choi et al., 2012). Further, it was recently shown that infusion of orexin-A into the pPVT facilitates cue-elicited behavioral responses for a food reward and concurrent neuronal activity in the NAcC in sated rats (Meffre et al., 2019). In the same report, it was shown that optogenetic stimulation of the pPVT elicits similar behavioral and neuronal results (Meffre et al., 2019). Others have reported that targeted optogenetic stimulation of the aPVT-NAcSh pathway also enhances motivation to consume food in hungry mice (Cheng et al., 2018), presumably *via* dopamine release in the NAcSh. These findings are consistent with those of Choudhary et al. (2018), who reported that food-seeking behavior in sated rats was impacted by CART signaling in LH-PVT projection neurons and, in turn, by glutamatergic PVT-NAcSh neurons. Similarly, activation of neurons in the PVT that express glucose transporter 2 and project to the NAc results in increased sucrose-seeking behavior, highlighting a potential role for this neuronal pathway in maintaining a balance between homeostatic and hedonic control of food intake (Labouèbe et al., 2016). More recently, it was shown that optogenetic stimulation of the PVT-NAcSh pathway is itself reinforcing; yet inhibition of the same pathway elicits responding for a food reward even when it is not available (Lafferty et al., 2020). Thus, the PVT-NAcSh pathway appears to promote efficient reward-seeking behavior. Taken together, these findings support the notion that the PVT acts to integrate information about intrinsic motivational states and the external environment, and, in turn, communicate with the NAc to guide consummatory and reward-seeking behaviors in an adaptive manner.

Beyond a specified role in reward-seeking behaviors, a number of reports suggest a more general role for the hypothalamic-thalamic-striatal circuit, and the PVT in particular, in mediating adaptive responses to both appetitive and aversive stimuli. In support, neurons in the PVT were found to respond similarly to stimuli associated with reward or punishment, with the neural response dependent on the intensity of the stimulus rather than the valence (Zhu et al., 2018). Further, PVT activity during Pavlovian cue presentation was shown to be required for both associative reward and aversive learning, as inhibition of PVT neurons decreases behavioral indices of a learned response to both positive and negative stimuli (Zhu et al., 2018). The seemingly indiscriminate encoding of the value of appetitive and aversive stimuli within the PVT supports the proposed role for this nucleus as a mediator of motivational conflict (McNally, 2021). McNally and colleagues have demonstrated that the PVT is necessary for enabling adaptive responses under conflicting behavioral tendencies toward danger and reward (Choi and McNally, 2017; Choi et al., 2019). Specifically, chemogenetic inhibition across the anterior-posterior axis of the PVT disrupts cue-elicited appetitive behavior and increases cue-elicited aversive behavior under conditions of motivational conflict (Choi et al., 2019). More recently, it was shown that a subpopulation of neurons in the aPVT that express corticotropin-releasing factor (CRF) is particularly important for regulating responses during motivational conflict (Engelke et al., 2021). Inactivation of these neurons biases an animal’s response toward food, whereas activation enables suppression of food-seeking behavior during conflict. Further, it was determined that input from the ventromedial hypothalamus to aPVT CRF neurons, and output from aPVT CRF neurons to the NAc, are critical components of the circuit that modulates reward-seeking behavior under competing demands of avoiding threats (Engelke et al., 2021). These studies collectively support a role for the PVT as an arbitrator that encodes the value of external stimuli and internal states and, in turn, facilitates adaptive behavior.

## A Proposed Role for The LH-PVT-NAc Circuit in Incentive Motivational Processes

Many studies implicating the PVT in motivated behavior have relied on associative learning paradigms. In fact, Ann Kelley and colleagues (Schiltz et al., 2007) identified the PVT as a “hot spot” as indexed by immediate early gene expression in response to stimuli associated with food reward (Schiltz et al., 2007), and a number of subsequent studies demonstrated that reward-associated cues and contexts similarly engage the PVT (Hamlin et al., 2009; Choi et al., 2010; Igelstrom et al., 2010). Recently, Otis and colleagues exploited new technologies to elucidate the circuits that facilitate cue-reactivity in the PVT (Otis et al., 2019). Their findings suggest that cue-reward information is transmitted *via* inhibitory responses in the PVT-NAc pathway and that this information is differentially encoded by input to the PVT from the prefrontal cortex (PFC) and LH. Specifically, they report that information about the cue-reward association is carried by glutamatergic axons from the PFC, whereas consummatory (i.e., licking) information is carried by GABAergic input from the LH (Otis et al., 2019). These findings support the hypothesis put forth by Ann Kelley and colleagues (Kelley et al., 2005) regarding the role of the LH-PVT-NAc axis in motivated behavior, and allow us to refine it. One caveat to such elegant technical approaches, however, is the inability to exploit behavioral output as an index of neural activity and function. That is, such approaches often require head-fixed animals or restraint in some manner that impedes behavior. Further, in this (Otis et al., 2019) and in fact, most studies assessing associative cue-reward relationships, it is difficult, if not impossible, to parse the value attributed to the cue. To circumvent this issue and better study the neural processes underlying cue-reward learning, we have exploited natural variation in cue-motivated behavior using a rat model, as described below [see also: Flagel and Robinson (2017) and Robinson and Flagel (2009)].

**Figure 2:**
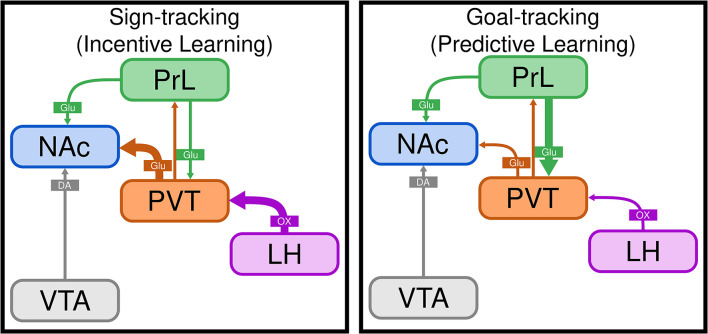
The PVT differentially mediates sign- and goal-tracking behavior. Schematic illustrating the paraventricular nucleus of the thalamus (PVT) as a central locus that acts to differentially regulate sign-tracking and goal-tracking behavior. Sign-tracking is a result of incentive cue-reward learning, whereas goal-tracking is the result of predictive cue-reward learning. We hypothesize that the incentive value of reward cues is encoded in the LH-PVT-NAc circuit (as indicated by thick purple and orange arrows), which is engaged to a greater degree in sign-trackers. In contrast, goal-trackers rely on top-down cortical control mechanisms (as indicated by thick green arrow) to encode the predictive value of reward cues and inhibit incentive motivational processes. LH, lateral hypothalamus; NAc, nucleus accumbens; PrL, prelimbic cortex; PVT, paraventricular thalamic nucleus; VTA, ventral tegmental area; DA, dopamine; Glu, glutamate; OX, orexin.

When rats are exposed to a Pavlovian conditioned approach (PavCA) paradigm, wherein presentation of a discrete cue (e.g., appearance of a lever) is followed by delivery of a food reward, distinct conditioned responses emerge (Robinson and Flagel, 2009). Upon cue presentation, some rats, sign-trackers (STs), learn to approach and interact with the cue itself; whereas others, goal-trackers (GTs) learn to go to the food cup and await reward delivery. The cue acquires predictive value for both STs and GTs, as it elicits a conditioned response for both (Robinson and Flagel, 2009). However, for STs, the cue also acquires incentive value (i.e., incentive salience) and is thereby transformed into a “motivational magnet” that is in and of itself attractive and desirable (Robinson and Berridge, 2001; Berridge and Robinson, 2003; Robinson and Flagel, 2009). Thus, the ST/GT model provides a unique opportunity to dissociate the neurobiological mechanisms that underlie predictive vs. incentive cue-reward learning; the latter of which is thought to be especially relevant to addiction and related disorders (Stewart et al., 1984; Childress et al., 1993; Robinson and Berridge, 1993). Using this model, we have identified the PVT as a critical node that mediates the propensity to attribute incentive motivational value to reward cues (Haight and Flagel, 2014; Haight et al., 2015, 2017; Kuhn et al., 2018a,b; Campus et al., 2019). Relative to GTs, STs show greater neuronal activity (c-Fos) in the PVT in response to both food- and drug-associated cues (Flagel et al., 2011a; Yager and Robinson, 2013). Cue-induced c-Fos mRNA levels in the PVT are correlated with those in a number of subcortical regions for STs, and cortical regions for GTs (Flagel et al., 2011a; Haight and Flagel, 2014). Further, there is greater cue-induced neuronal activity (c-Fos) specifically in LH-PVT and PVT-NAc projection neurons in STs relative to GTs (Haight et al., 2017). To further investigate the role of the LH-PVT pathway in incentive learning, we examined the effects of lesioning the LH and blocking orexinergic activity in the PVT. We found that LH lesions block the *development* of sign-tracking behavior, and administration of OX-1 or OX-2 antagonists into the PVT attenuates the *expression* of sign-tracking behavior (Haight et al., 2020). Together, these data led us to develop a neural model of sign-tracking (Figure 2); whereby orexinergic transmission from the LH-PVT encodes the incentive value of cues, altering communication in the PVT-NAcSh pathway, and subsequently, dopamine activity, which we know is critical for incentive learning (Flagel et al., 2011b). In contrast, we have data to suggest that goal-tracking behavior is facilitated *via* a cortico-thalamic-striatal pathway (see Figure 2), with input from the PrL to the PVT encoding the predictive value of reward cues and thereby modulating output from the PVT-NAc to promote goal-directed behavior (Campus et al., 2019). Together, these findings point to the PVT as a critical node in the regulation of distinct cue-reward learning strategies, with the LH-PVT-NAc circuit playing a specific role in incentive motivational processes.

## Conclusion

In merging the last decade of behavioral neuroscience research surrounding the PVT, we have provided an outline of how this midline thalamic nucleus acts as a critical node in motivated behavior. The PVT was appropriately interwoven within the classic reward circuitry by Kelley and colleagues almost 20 years ago (Kelley et al., 2005). Various anatomical, pharmacological, and behavioral studies have since implicated the PVT in emotional valence, appetitive and aversive motivation, and behavioral regulation. As such, the PVT has earned its spot in the motive circuit, acting to multiplex interoceptive and exteroceptive cues to adaptively guide motivated behavior. Using state-of-the-art technology, we can now probe the role of the PVT and its associated circuitry more deeply and more precisely than ever before. Indeed, such approaches have allowed us to refine the original hypothesis put forth by Kelley and colleagues, and ascribe more specific roles to targeted pathways and neurotransmitter systems within PVT circuitry. Further, we have highlighted how behavioral approaches, like those exploiting inherent individual variability, allow us to parse the role of the PVT in specific forms of cue-reward learning. Together, this body of work has led us to postulate that the PVT dynamically regulates individual differences in associative learning processes, with the LH-PVT-NAc circuit acting to encode the incentive value of reward-cues. This latter process enables environmental stimuli to attain inordinate control over behavior and lead to maladaptive behavior reminiscent of that which characterizes addiction and impulse control disorders. Thus, in conjunction with its role in “motivated behavior”, we believe the PVT—*via* its connections to both cortical and subcortical regions—plays a critical role in modulating the affective and behavioral patterns that underlie shared facets of these and other psychiatric conditions.

## Data Availability Statement

The original contributions presented in the study are included in the article, further inquiries can be directed to the corresponding author.

## Author Contributions

AI and SF wrote the manuscript together. All authors contributed to the article and approved the submitted version.

## Conflict of Interest

The authors declare that the research was conducted in the absence of any commercial or financial relationships that could be construed as a potential conflict of interest.
